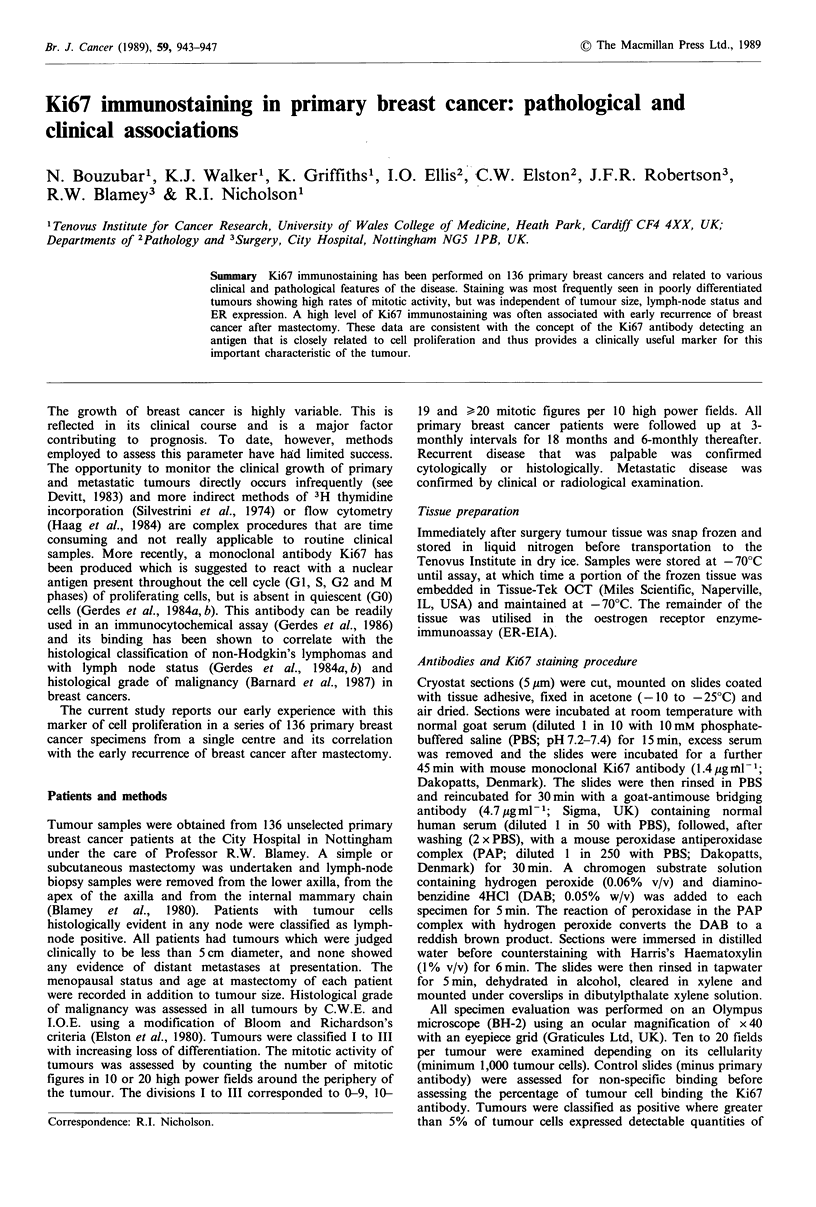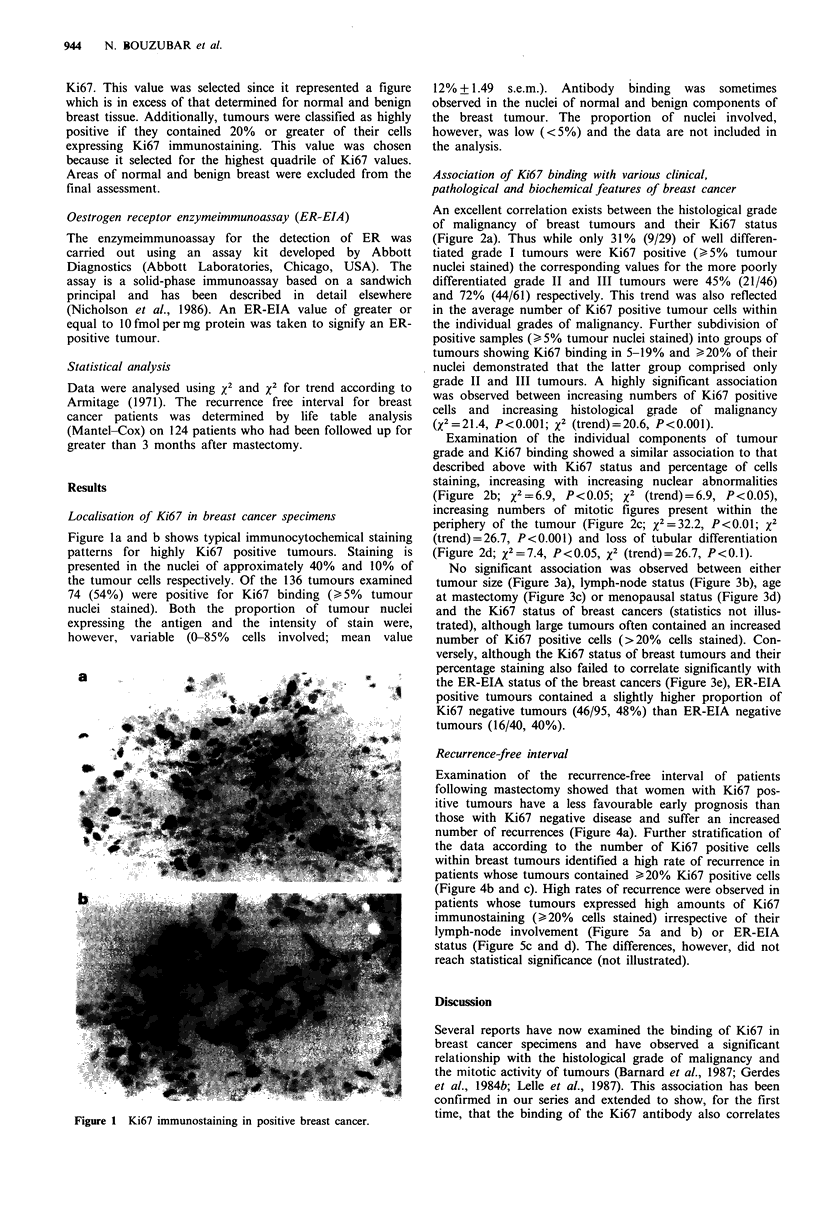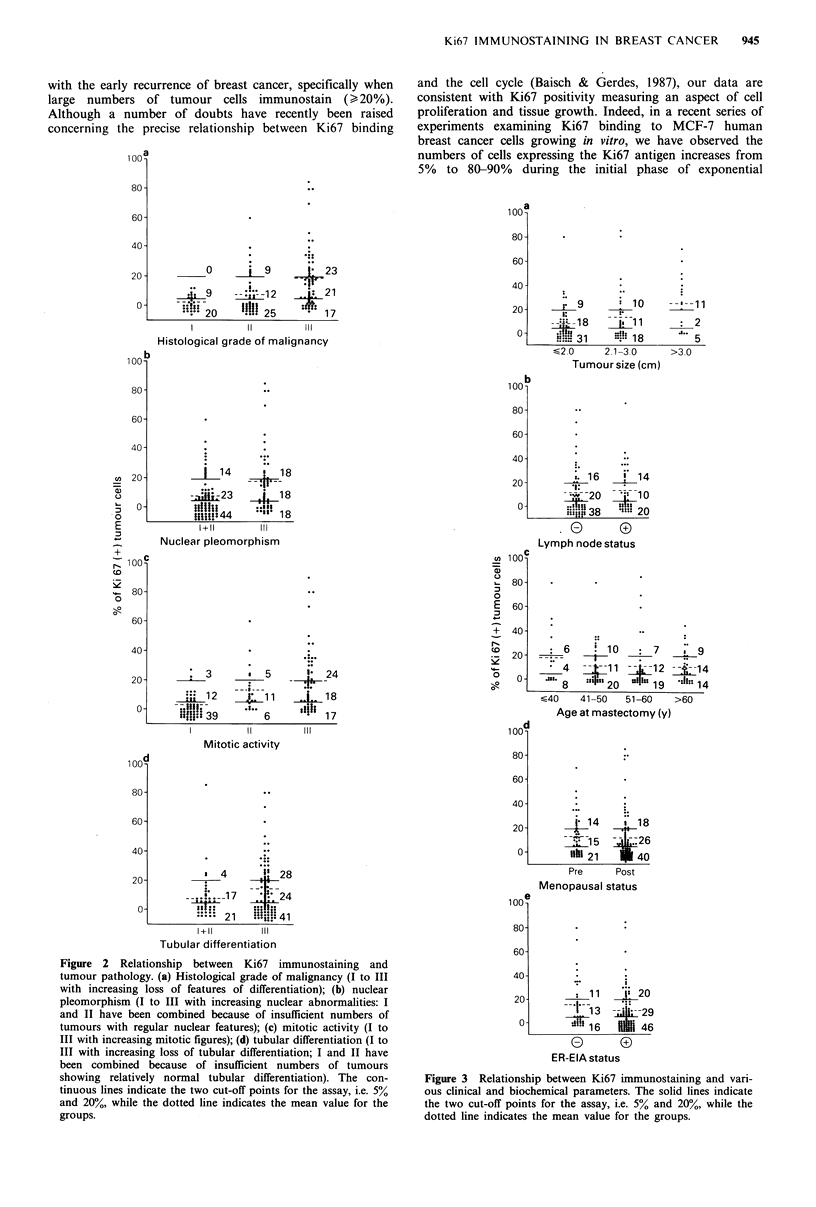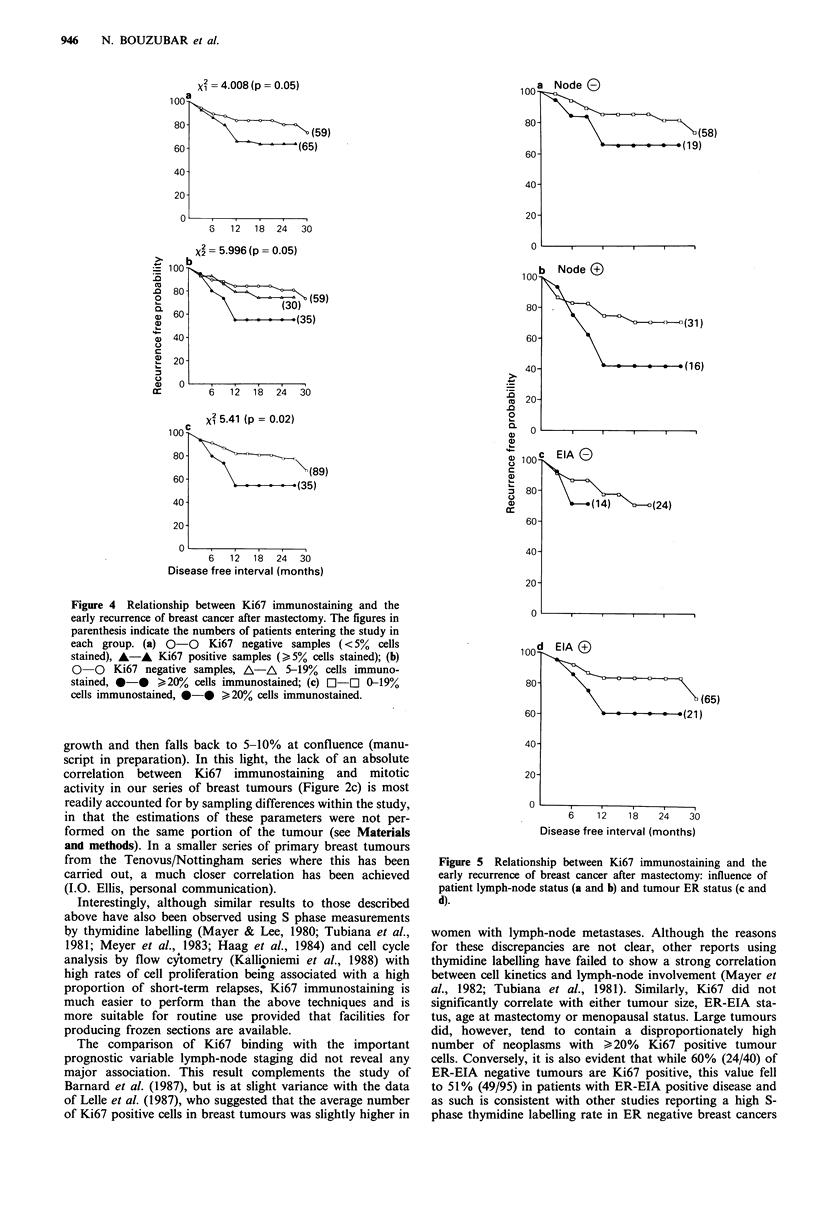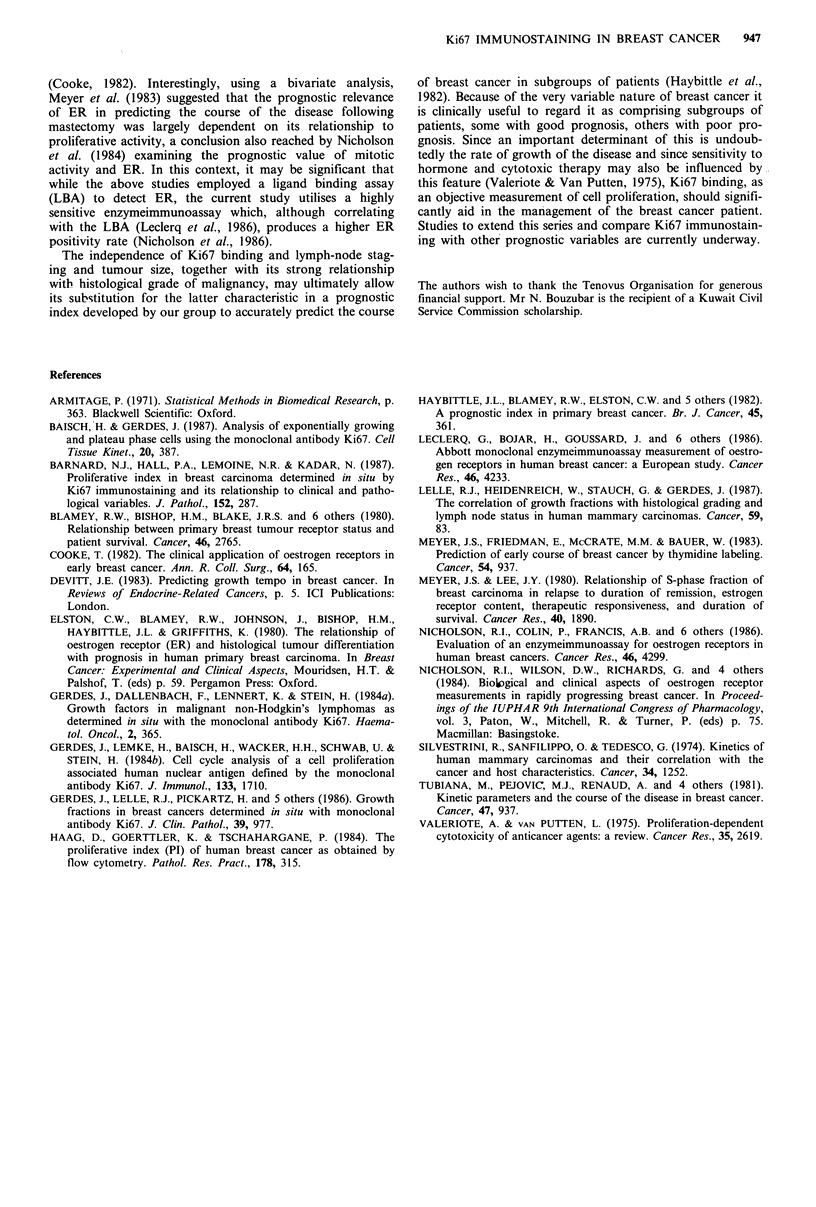# Ki67 immunostaining in primary breast cancer: pathological and clinical associations.

**DOI:** 10.1038/bjc.1989.200

**Published:** 1989-06

**Authors:** N. Bouzubar, K. J. Walker, K. Griffiths, I. O. Ellis, C. W. Elston, J. F. Robertson, R. W. Blamey, R. I. Nicholson

**Affiliations:** Tenovus Institute for Cancer Research, University of Wales College of Medicine, Health Park, Cardiff, UK.

## Abstract

**Images:**


*Br. J. Cancer* (1989), **59**, 943–947

© The Macmillan Press Ltd., 1989

### Ki67 immunostaining in primary breast cancer: pathological and clinical associations

N. Bouzubar[1], K.J. Walker[1], K. Griffiths[1], I.O. Ellis[2], C.W. Elston[2], J.F.R. Robertson[3], R.W. Blamey[3] & R.I. Nicholson[1]

[1]*Tenovus Institute for Cancer Research, University of Wales College of Medicine, Heath Park, Cardiff CF4 4XX, UK; Departments of* [2]*Pathology and* [3]*Surgery, City Hospital, Nottingham NG5 1PB, UK.*

**Summary** Ki67 immunostaining has been performed on 136 primary breast cancers and related to various clinical and pathological features of the disease. Staining was most frequently seen in poorly differentiated tumours showing high rates of mitotic activity, but was independent of tumour size, lymph-node status and ER expression. A high level of Ki67 immunostaining was often associated with early recurrence of breast cancer after mastectomy. These data are consistent with the concept of the Ki67 antibody detecting an antigen that is closely related to cell proliferation and thus provides a clinically useful marker for this important characteristic of the tumour.

The growth of breast cancer is highly variable. This is reflected in its clinical course and is a major factor contributing to prognosis. To date, however, methods employed to assess this parameter have had limited success. The opportunity to monitor the clinical growth of primary and metastatic tumours directly occurs infrequently (see Devitt, 1983) and more indirect methods of $^3$H thymidine incorporation (Silvestrini *et al.*, 1974) or flow cytometry (Haag *et al.*, 1984) are complex procedures that are time consuming and not really applicable to routine clinical samples. More recently, a monoclonal antibody Ki67 has been produced which is suggested to react with a nuclear antigen present throughout the cell cycle (G1, S, G2 and M phases) of proliferating cells, but is absent in quiescent (G0) cells (Gerdes *et al.*, 1984*a*, *b*). This antibody can be readily used in an immunocytochemical assay (Gerdes *et al.*, 1986) and its binding has been shown to correlate with the histological classification of non-Hodgkin's lymphomas and with lymph node status (Gerdes *et al.*, 1984*a*, *b*) and histological grade of malignancy (Barnard *et al.*, 1987) in breast cancers.

The current study reports our early experience with this marker of cell proliferation in a series of 136 primary breast cancer specimens from a single centre and its correlation with the early recurrence of breast cancer after mastectomy.

### Patients and methods

Tumour samples were obtained from 136 unselected primary breast cancer patients at the City Hospital in Nottingham under the care of Professor R.W. Blamey. A simple or subcutaneous mastectomy was undertaken and lymph-node biopsy samples were removed from the lower axilla, from the apex of the axilla and from the internal mammary chain (Blamey *et al.*, 1980). Patients with tumour cells histologically evident in any node were classified as lymph-node positive. All patients had tumours which were judged clinically to be less than 5 cm diameter, and none showed any evidence of distant metastases at presentation. The menopausal status and age at mastectomy of each patient were recorded in addition to tumour size. Histological grade of malignancy was assessed in all tumours by C.W.E. and I.O.E. using a modification of Bloom and Richardson's criteria (Elston *et al.*, 1980). Tumours were classified I to III with increasing loss of differentiation. The mitotic activity of tumours was assessed by counting the number of mitotic figures in 10 or 20 high power fields around the periphery of the tumour. The divisions I to III corresponded to 0–9, 10–19 and ≥20 mitotic figures per 10 high power fields. All primary breast cancer patients were followed up at 3-monthly intervals for 18 months and 6-monthly thereafter. Recurrent disease that was palpable was confirmed cytologically or histologically. Metastatic disease was confirmed by clinical or radiological examination.

Correspondence: R.I. Nicholson.

### *Tissue preparation*

Immediately after surgery tumour tissue was snap frozen and stored in liquid nitrogen before transportation to the Tenovus Institute in dry ice. Samples were stored at −70°C until assay, at which time a portion of the frozen tissue was embedded in Tissue-Tek OCT (Miles Scientific, Naperville, IL, USA) and maintained at −70°C. The remainder of the tissue was utilised in the oestrogen receptor enzyme-immunoassay (ER-EIA).

### *Antibodies and Ki67 staining procedure*

Cryostat sections (5 μm) were cut, mounted on slides coated with tissue adhesive, fixed in acetone (−10 to −25°C) and air dried. Sections were incubated at room temperature with normal goat serum (diluted 1 in 10 with 10 mM phosphate-buffered saline (PBS; pH 7.2–7.4) for 15 min, excess serum was removed and the slides were incubated for a further 45 min with mouse monoclonal Ki67 antibody (1.4 $\mu g\,ml^{-1}$; Dakopatts, Denmark). The slides were then rinsed in PBS and reincubated for 30 min with a goat-antimouse bridging antibody (4.7 $\mu g\,ml^{-1}$; Sigma, UK) containing normal human serum (diluted 1 in 50 with PBS), followed, after washing (2 × PBS), with a mouse peroxidase antiperoxidase complex (PAP; diluted 1 in 250 with PBS; Dakopatts, Denmark) for 30 min. A chromogen substrate solution containing hydrogen peroxide (0.06% v/v) and diamino-benzidine 4HCl (DAB; 0.05% w/v) was added to each specimen for 5 min. The reaction of peroxidase in the PAP complex with hydrogen peroxide converts the DAB to a reddish brown product. Sections were immersed in distilled water before counterstaining with Harris's Haematoxylin (1% v/v) for 6 min. The slides were then rinsed in tapwater for 5 min, dehydrated in alcohol, cleared in xylene and mounted under coverslips in dibutylpthalate xylene solution.

All specimen evaluation was performed on an Olympus microscope (BH-2) using an ocular magnification of ×40 with an eyepiece grid (Graticules Ltd, UK). Ten to 20 fields per tumour were examined depending on its cellularity (minimum 1,000 tumour cells). Control slides (minus primary antibody) were assessed for non-specific binding before assessing the percentage of tumour cell binding the Ki67 antibody. Tumours were classified as positive where greater than 5% of tumour cells expressed detectable quantities of

Ki67. This value was selected since it represented a figure which is in excess of that determined for normal and benign breast tissue. Additionally, tumours were classified as highly positive if they contained 20% or greater of their cells expressing Ki67 immunostaining. This value was chosen because it selected for the highest quadrile of Ki67 values. Areas of normal and benign breast were excluded from the final assessment.

*Oestrogen receptor enzymeimmunoassay (ER-EIA)*

The enzymeimmunoassay for the detection of ER was carried out using an assay kit developed by Abbott Diagnostics (Abbott Laboratories, Chicago, USA). The assay is a solid-phase immunoassay based on a sandwich principal and has been described in detail elsewhere (Nicholson *et al.*, 1986). An ER-EIA value of greater or equal to 10 fmol per mg protein was taken to signify an ER-positive tumour.

*Statistical analysis*

Data were analysed using $\chi^2$ and $\chi^2$ for trend according to Armitage (1971). The recurrence free interval for breast cancer patients was determined by life table analysis (Mantel–Cox) on 124 patients who had been followed up for greater than 3 months after mastectomy.

### Results

*Localisation of Ki67 in breast cancer specimens*

Figure 1a and b shows typical immunocytochemical staining patterns for highly Ki67 positive tumours. Staining is presented in the nuclei of approximately 40% and 10% of the tumour cells respectively. Of the 136 tumours examined 74 (54%) were positive for Ki67 binding ($\geqslant$5% tumour nuclei stained). Both the proportion of tumour nuclei expressing the antigen and the intensity of stain were, however, variable (0–85% cells involved; mean value 12% $\pm$ 1.49 s.e.m.). Antibody binding was sometimes observed in the nuclei of normal and benign components of the breast tumour. The proportion of nuclei involved, however, was low (<5%) and the data are not included in the analysis.

**Figure 1** Ki67 immunostaining in positive breast cancer.

*Association of Ki67 binding with various clinical, pathological and biochemical features of breast cancer*

An excellent correlation exists between the histological grade of malignancy of breast tumours and their Ki67 status (Figure 2a). Thus while only 31% (9/29) of well differentiated grade I tumours were Ki67 positive ($\geqslant$5% tumour nuclei stained) the corresponding values for the more poorly differentiated grade II and III tumours were 45% (21/46) and 72% (44/61) respectively. This trend was also reflected in the average number of Ki67 positive tumour cells within the individual grades of malignancy. Further subdivision of positive samples ($\geqslant$5% tumour nuclei stained) into groups of tumours showing Ki67 binding in 5–19% and $\geqslant$20% of their nuclei demonstrated that the latter group comprised only grade II and III tumours. A highly significant association was observed between increasing numbers of Ki67 positive cells and increasing histological grade of malignancy ($\chi^2=21.4$, $P<0.001$; $\chi^2$ (trend)$=20.6$, $P<0.001$).

Examination of the individual components of tumour grade and Ki67 binding showed a similar association to that described above with Ki67 status and percentage of cells staining, increasing with increasing nuclear abnormalities (Figure 2b; $\chi^2=6.9$, $P<0.05$; $\chi^2$ (trend)$=6.9$, $P<0.05$), increasing numbers of mitotic figures present within the periphery of the tumour (Figure 2c; $\chi^2=32.2$, $P<0.01$; $\chi^2$ (trend)$=26.7$, $P<0.001$) and loss of tubular differentiation (Figure 2d; $\chi^2=7.4$, $P<0.05$, $\chi^2$ (trend)$=26.7$, $P<0.1$).

No significant association was observed between either tumour size (Figure 3a), lymph-node status (Figure 3b), age at mastectomy (Figure 3c) or menopausal status (Figure 3d) and the Ki67 status of breast cancers (statistics not illustrated), although large tumours often contained an increased number of Ki67 positive cells (>20% cells stained). Conversely, although the Ki67 status of breast tumours and their percentage staining also failed to correlate significantly with the ER-EIA status of the breast cancers (Figure 3e), ER-EIA positive tumours contained a slightly higher proportion of Ki67 negative tumours (46/95, 48%) than ER-EIA negative tumours (16/40, 40%).

*Recurrence-free interval*

Examination of the recurrence-free interval of patients following mastectomy showed that women with Ki67 positive tumours have a less favourable early prognosis than those with Ki67 negative disease and suffer an increased number of recurrences (Figure 4a). Further stratification of the data according to the number of Ki67 positive cells within breast tumours identified a high rate of recurrence in patients whose tumours contained $\geqslant$20% Ki67 positive cells (Figure 4b and c). High rates of recurrence were observed in patients whose tumours expressed high amounts of Ki67 immunostaining ($\geqslant$20% cells stained) irrespective of their lymph-node involvement (Figure 5a and b) or ER-EIA status (Figure 5c and d). The differences, however, did not reach statistical significance (not illustrated).

### Discussion

Several reports have now examined the binding of Ki67 in breast cancer specimens and have observed a significant relationship with the histological grade of malignancy and the mitotic activity of tumours (Barnard *et al.*, 1987; Gerdes *et al.*, 1984*b*; Lelle *et al.*, 1987). This association has been confirmed in our series and extended to show, for the first time, that the binding of the Ki67 antibody also correlates with the early recurrence of breast cancer, specifically when large numbers of tumour cells immunostain (≥20%). Although a number of doubts have recently been raised concerning the precise relationship between Ki67 binding and the cell cycle (Baisch & Gerdes, 1987), our data are consistent with Ki67 positivity measuring an aspect of cell proliferation and tissue growth. Indeed, in a recent series of experiments examining Ki67 binding to MCF-7 human breast cancer cells growing *in vitro*, we have observed the numbers of cells expressing the Ki67 antigen increases from 5% to 80–90% during the initial phase of exponential

**Figure 2** Relationship between Ki67 immunostaining and tumour pathology. (**a**) Histological grade of malignancy (I to III with increasing loss of features of differentiation); (**b**) nuclear pleomorphism (I to III with increasing nuclear abnormalities: I and II have been combined because of insufficient numbers of tumours with regular nuclear features); (**c**) mitotic activity (I to III with increasing mitotic figures); (**d**) tubular differentiation (I to III with increasing loss of tubular differentiation; I and II have been combined because of insufficient numbers of tumours showing relatively normal tubular differentiation). The continuous lines indicate the two cut-off points for the assay, i.e. 5% and 20%, while the dotted line indicates the mean value for the groups.

**Figure 3** Relationship between Ki67 immunostaining and various clinical and biochemical parameters. The solid lines indicate the two cut-off points for the assay, i.e. 5% and 20%, while the dotted line indicates the mean value for the groups.

**Figure 4** Relationship between Ki67 immunostaining and the early recurrence of breast cancer after mastectomy. The figures in parenthesis indicate the numbers of patients entering the study in each group. (**a**) O—O Ki67 negative samples (<5% cells stained), ▲—▲ Ki67 positive samples (≥5% cells stained); (**b**) O—O Ki67 negative samples, △—△ 5–19% cells immunostained, ●—● ≥20% cells immunostained; (**c**) □—□ 0–19% cells immunostained, ●—● ≥20% cells immunostained.

**Figure 5** Relationship between Ki67 immunostaining and the early recurrence of breast cancer after mastectomy: influence of patient lymph-node status (**a** and **b**) and tumour ER status (**c** and **d**).

growth and then falls back to 5–10% at confluence (manuscript in preparation). In this light, the lack of an absolute correlation between Ki67 immunostaining and mitotic activity in our series of breast tumours (Figure 2c) is most readily accounted for by sampling differences within the study, in that the estimations of these parameters were not performed on the same portion of the tumour (see **Materials and methods**). In a smaller series of primary breast tumours from the Tenovus/Nottingham series where this has been carried out, a much closer correlation has been achieved (I.O. Ellis, personal communication).

Interestingly, although similar results to those described above have also been observed using S phase measurements by thymidine labelling (Mayer & Lee, 1980; Tubiana *et al.*, 1981; Meyer *et al.*, 1983; Haag *et al.*, 1984) and cell cycle analysis by flow cytometry (Kallioniemi *et al.*, 1988) with high rates of cell proliferation being associated with a high proportion of short-term relapses, Ki67 immunostaining is much easier to perform than the above techniques and is more suitable for routine use provided that facilities for producing frozen sections are available.

The comparison of Ki67 binding with the important prognostic variable lymph-node staging did not reveal any major association. This result complements the study of Barnard *et al.* (1987), but is at slight variance with the data of Lelle *et al.* (1987), who suggested that the average number of Ki67 positive cells in breast tumours was slightly higher in women with lymph-node metastases. Although the reasons for these discrepancies are not clear, other reports using thymidine labelling have failed to show a strong correlation between cell kinetics and lymph-node involvement (Mayer *et al.*, 1982; Tubiana *et al.*, 1981). Similarly, Ki67 did not significantly correlate with either tumour size, ER-EIA status, age at mastectomy or menopausal status. Large tumours did, however, tend to contain a disproportionately high number of neoplasms with ≥20% Ki67 positive tumour cells. Conversely, it is also evident that while 60% (24/40) of ER-EIA negative tumours are Ki67 positive, this value fell to 51% (49/95) in patients with ER-EIA positive disease and as such is consistent with other studies reporting a high S-phase thymidine labelling rate in ER negative breast cancers

(Cooke, 1982). Interestingly, using a bivariate analysis, Meyer *et al.* (1983) suggested that the prognostic relevance of ER in predicting the course of the disease following mastectomy was largely dependent on its relationship to proliferative activity, a conclusion also reached by Nicholson *et al.* (1984) examining the prognostic value of mitotic activity and ER. In this context, it may be significant that while the above studies employed a ligand binding assay (LBA) to detect ER, the current study utilises a highly sensitive enzymeimmunoassay which, although correlating with the LBA (Leclerq *et al.*, 1986), produces a higher ER positivity rate (Nicholson *et al.*, 1986).

The independence of Ki67 binding and lymph-node staging and tumour size, together with its strong relationship with histological grade of malignancy, may ultimately allow its substitution for the latter characteristic in a prognostic index developed by our group to accurately predict the course of breast cancer in subgroups of patients (Haybittle *et al.*, 1982). Because of the very variable nature of breast cancer it is clinically useful to regard it as comprising subgroups of patients, some with good prognosis, others with poor prognosis. Since an important determinant of this is undoubtedly the rate of growth of the disease and since sensitivity to hormone and cytotoxic therapy may also be influenced by this feature (Valeriote & Van Putten, 1975), Ki67 binding, as an objective measurement of cell proliferation, should significantly aid in the management of the breast cancer patient. Studies to extend this series and compare Ki67 immunostaining with other prognostic variables are currently underway.

The authors wish to thank the Tenovus Organisation for generous financial support. Mr N. Bouzubar is the recipient of a Kuwait Civil Service Commission scholarship.

**References**

ARMITAGE, P. (1971). *Statistical Methods in Biomedical Research*, p. 363. Blackwell Scientific: Oxford.

BAISCH, H. & GERDES, J. (1987). Analysis of exponentially growing and plateau phase cells using the monoclonal antibody Ki67. *Cell Tissue Kinet.*, **20**, 387.

BARNARD, N.J., HALL, P.A., LEMOINE, N.R. & KADAR, N. (1987). Proliferative index in breast carcinoma determined *in situ* by Ki67 immunostaining and its relationship to clinical and pathological variables. *J. Pathol.*, **152**, 287.

BLAMEY, R.W., BISHOP, H.M., BLAKE, J.R.S. and 6 others (1980). Relationship between primary breast tumour receptor status and patient survival. *Cancer*, **46**, 2765.

COOKE, T. (1982). The clinical application of oestrogen receptors in early breast cancer. *Ann. R. Coll. Surg.*, **64**, 165.

DEVITT, J.E. (1983). Predicting growth tempo in breast cancer. In *Reviews of Endocrine-Related Cancers*, p. 5. ICI Publications: London.

ELSTON, C.W., BLAMEY, R.W., JOHNSON, J., BISHOP, H.M., HAYBITTLE, J.L. & GRIFFITHS, K. (1980). The relationship of oestrogen receptor (ER) and histological tumour differentiation with prognosis in human primary breast carcinoma. In *Breast Cancer: Experimental and Clinical Aspects*, Mouridsen, H.T. & Palshof, T. (eds) p. 59. Pergamon Press: Oxford.

GERDES, J., DALLENBACH, F., LENNERT, K. & STEIN, H. (1984*a*). Growth factors in malignant non-Hodgkin's lymphomas as determined *in situ* with the monoclonal antibody Ki67. *Haematol. Oncol.*, **2**, 365.

GERDES, J., LEMKE, H., BAISCH, H., WACKER, H.H., SCHWAB, U. & STEIN, H. (1984*b*). Cell cycle analysis of a cell proliferation associated human nuclear antigen defined by the monoclonal antibody Ki67. *J. Immunol.*, **133**, 1710.

GERDES, J., LELLE, R.J., PICKARTZ, H. and 5 others (1986). Growth fractions in breast cancers determined *in situ* with monoclonal antibody Ki67. *J. Clin. Pathol.*, **39**, 977.

HAAG, D., GOERTTLER, K. & TSCHAHARGANE, P. (1984). The proliferative index (PI) of human breast cancer as obtained by flow cytometry. *Pathol. Res. Pract.*, **178**, 315.

HAYBITTLE, J.L., BLAMEY, R.W., ELSTON, C.W. and 5 others (1982). A prognostic index in primary breast cancer. *Br. J. Cancer*, **45**, 361.

LECLERQ, G., BOJAR, H., GOUSSARD, J. and 6 others (1986). Abbott monoclonal enzymeimmunoassay measurement of oestrogen receptors in human breast cancer: a European study. *Cancer Res.*, **46**, 4233.

LELLE, R.J., HEIDENREICH, W., STAUCH, G. & GERDES, J. (1987). The correlation of growth fractions with histological grading and lymph node status in human mammary carcinomas. *Cancer*, **59**, 83.

MEYER, J.S., FRIEDMAN, E., MCCRATE, M.M. & BAUER, W. (1983). Prediction of early course of breast cancer by thymidine labeling. *Cancer*, **54**, 937.

MEYER, J.S. & LEE, J.Y. (1980). Relationship of S-phase fraction of breast carcinoma in relapse to duration of remission, estrogen receptor content, therapeutic responsiveness, and duration of survival. *Cancer Res.*, **40**, 1890.

NICHOLSON, R.I., COLIN, P., FRANCIS, A.B. and 6 others (1986). Evaluation of an enzymeimmunoassay for oestrogen receptors in human breast cancers. *Cancer Res.*, **46**, 4299.

NICHOLSON, R.I., WILSON, D.W., RICHARDS, G. and 4 others (1984). Biological and clinical aspects of oestrogen receptor measurements in rapidly progressing breast cancer. In *Proceedings of the IUPHAR 9th International Congress of Pharmacology*, vol. 3, Paton, W., Mitchell, R. & Turner, P. (eds) p. 75. Macmillan: Basingstoke.

SILVESTRINI, R., SANFILIPPO, O. & TEDESCO, G. (1974). Kinetics of human mammary carcinomas and their correlation with the cancer and host characteristics. *Cancer*, **34**, 1252.

TUBIANA, M., PEJOVIC, M.J., RENAUD, A. and 4 others (1981). Kinetic parameters and the course of the disease in breast cancer. *Cancer*, **47**, 937.

VALERIOTE, A. & VAN PUTTEN, L. (1975). Proliferation-dependent cytotoxicity of anticancer agents: a review. *Cancer Res.*, **35**, 2619.